# Enlargement of the Surface Area of High‐Entropy Alloys with Acid Treatment as Positive Electrode for High Specific Capacitance Supercapacitors

**DOI:** 10.1002/open.202400312

**Published:** 2025-01-26

**Authors:** Mahir Gülen, Recep Taş, Hamza Dünya, Shashanka Rajendrachari

**Affiliations:** ^1^ Bartin University Faculty of Engineering Architecture and Design Department of Mechanical Engineering 74100 Bartin Turkey; ^2^ Bartin University Faculty of Science Department of Biotechnology 74100 Bartin Turkey; ^3^ Department of Chemistry School of Sciences and Humanities SR University Warangal, Telangana 506371 India

**Keywords:** High Entropy Alloy (HEA), 23Fe−21Cr−18Ni−20Ti−18Mn, Supercapacitors, Acid Treatment

## Abstract

High‐entropy alloys (HEAs), containing five or more elements in equal proportions, have recently made significant achievements in materials science due to their remarkable properties, including high toughness, excellent catalytic, thermal, and electrical conductivity, and resistance to wear and corrosion. This study focuses on a HEA composed of 23Fe−21Cr−18Ni−20Ti−18Mn, synthesized via ball milling. The alloy was treated with hydrochloric acid (HCl) to enhance its active surface area. The untreated HEA and the HCl‐treated HEA (HEA‐T) were then evaluated as potential cathode materials for supercapacitors (SCs). X‐ray diffraction (XRD) and energy‐dispersive X‐ray spectroscopy (EDX) confirmed that the HEA's composition and crystalline structure remained stable during acid treatment, with no new phases forming. The acid treatment significantly increased the surface area by ~20 times, the pore volume by ~10 times, and improved microstructural homogeneity. The HEA‐T electrode demonstrated superior specific capacitance, lower internal resistance, and better cycling stability than the untreated HEA electrode. At 0.5 A/g current density, the specific capacitance (Csp) of the HEA‐T was 600 F/g, approximately two times higher than the untreated HEA. This enhanced performance suggests that the HEA‐T electrode could lead to the development of high‐performance SCs.

## Graphical abstract

A high entropy alloy (HEA) with 23Fe−21Cr−18Ni−20Ti−18Mn composition was synthesized using ball milling and treated with HCl acid to enhance supercapacitor performance. Acid treatment significantly improved the microstructure, increasing the surface area from 0.859 to 18.72 m^2^.g^−1^. Acid‐treated HEA exhibited 600 F/g of specific capacitance, 2.06 times higher than untreated HEA, showing stable performance at high current densities.

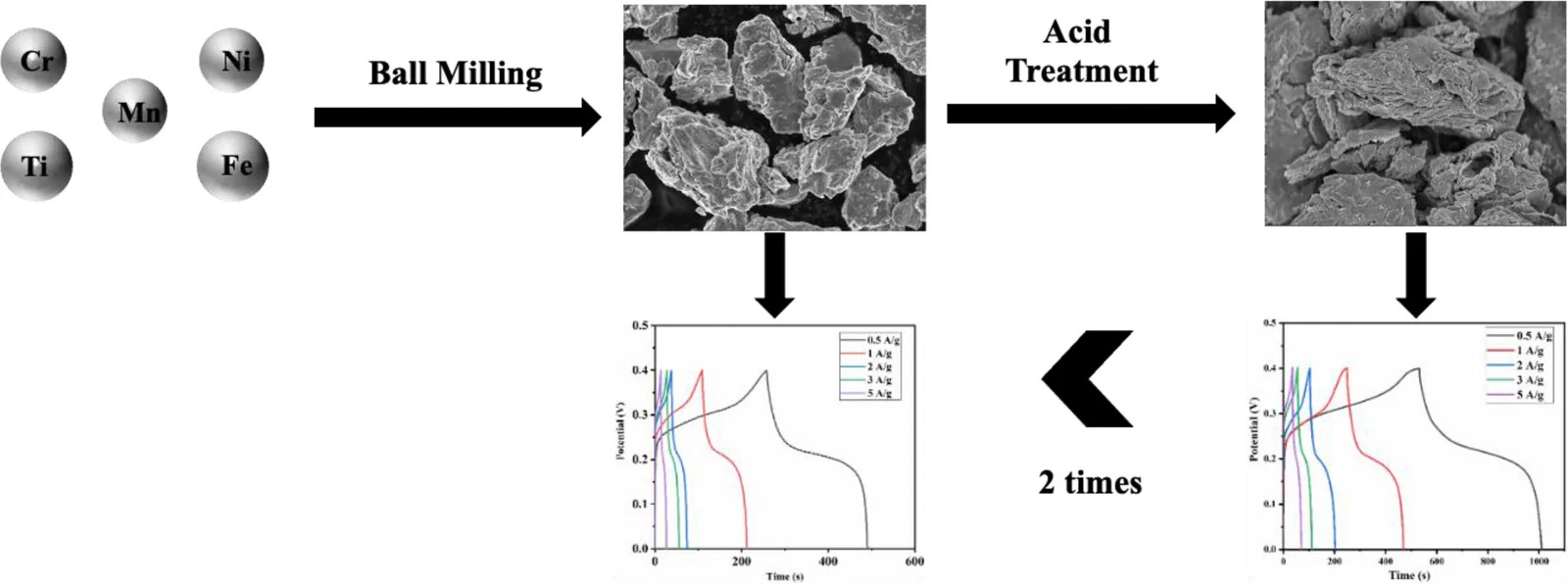

## 1 Introduction

The depletion of fossil fuel and running accumulation of greenhouse gasses push researchers to search for novel and greatly sustainable energy storage systems.^[1]^ To solve the problems of climate change and at the same time meet the urgent need for efficient energy storage, research is being carried out on energy storage devices such as batteries^[2]^ and supercapacitors,^[3]^ which have high energy and power density, are compatible with solar, wind and nuclear power harvester installations, are low‐cost and environmentally friendly.^[4]^ Among the energy storage technologies, supercapacitors (SCs) bridge the gap between conventional capacitors and batteries by offering higher energy densities than conventional capacitors and higher power densities than batteries. Furthermore, SCs when compared with other energy storage systems have long‐term cycle stability, high energy density, fast charge/discharge characteristics, elastic packaging, broad thermal range, quiet maintenance, and weight.^[5]^ This makes SCs ideal for applications requiring quick energy bursts and long‐term energy storage, such as in electric vehicles (EVs), portable electronics, and grid energy storage systems.^[6]^ The architecture of an SC includes an electrode, current collector, electrolyte, and separator. Among them, the electrode is the heart of SC and the center of effective transformation and storage of electrochemical energy. Therefore, the successful design and improvement of electrode materials have become vital for making up SCs with high performance.^[7]^ Numerous materials have been broadly examined as electrodes in SC.^[8]^ In general, metal oxide/sulphide/nitride/phosphide and conducting polymers have been used as electrode materials that exhibit high energy density, but low cycle life and fairly low power density. On the other hand, carbonous electrode materials demonstrate considerable cycle stability and fast charging‐discharging ability, but their poor energy density restricts practical applications.^[9]^ Despite the employment of several combinations of electrode materials in SCs, the resulting electrical conductivity, long‐term cycle stability, and high energy density at high power density are still insufficient for commercialization of SCs.^[10]^

The research in metallurgy has witnessed a remarkable development in recent decades due to the significant development in alloy design, fabrication methods, and extraordinary applications. Nowadays researchers are refining the microstructure, altering the compositions, incorporating the dispersants, and preparing multi‐elemental alloys with super properties and applications.^[11]^ One such super alloy is high entropy alloy (HEA); it is a combination of 5 or more elements with almost the same compositions by wt. %. These are the new grades of superalloys, that have significant properties, like high electrocatalytic activity, toughness, excellent thermal and electrical conductivity, wear and corrosion‐resistant properties. These super properties are due to their complex composition and the increased mixing entropy compared to the mixing entropy of bi, tri‐metallic, or pure metals.^[12]^ Due to this high mixing entropy, the enthalpy of the HEAs increases which then allows for the addition of a greater number of elements during fabrication.^[13]^ HEAs are considered to be the alternatives to various steel, stainless steel, copper, titanium, and aluminum alloys. In recent years, Shashanka et al. reported the excellent electrochemical properties of HEAs in determining ascorbic acids and methylene blue.^11^, ^14^ Furthermore, HEAs are frequently mentioned as promising materials for energy storage such as batteries,^[15]^ supercapacitors,^4^, ^16^ and hydrogen storage.^[17]^ It is emphasized that these materials have advantages such as high electrochemical stability, a wide potential range, and good cycle life. Unlike conventional alloys, HEAs are alloys containing five or more elements in equal proportions making HEAs a potential solution for energy storage applications.^[4]^ These properties make HEAs suitable for various advanced applications, including energy storage, catalysis, and structural materials.^[18]^ For example, Abid et al. reported the superior electrochemical performance of a CoCrFeNiMn HEA as an electrode material for supercapacitors, highlighting its high specific capacitance and excellent cycling stability.^[19]^ In another study, Kim et al., synthesized AlCoCrFeNi HEA, using the selective phase dissolution method and investigated its electrochemical properties as electrode material for SC. In the study, AlCoCrFeNi HEA electrode demonstrated high volumetric capacitance of 700 Fcm^−3^ and excellent cycling stability of over 3000 cycles.^[20]^ In addition, Sheng et al. synthesized high entropy metal nitride (including V, Cr, Nb, Mo, and Zr metals) via the Mechanochemical‐Assisted technique and employed it as a cathode in SC architecture. A specific capacitance of 78 F g^−1^ is accomplished at a scan rate of 100 mV s^−1^ in 1 M KOH.^[21]^ Similarly, other research has shown that the unique properties of HEAs, such as their high mechanical strength and excellent conductivity, can be harnessed to develop advanced energy storage devices.^[22]^ However, there is a lack of literature on the use of HEAs as electrodes in SCs.

A critical approach to enhance the electrocatalytic performance of electrode material is to enlarge the specific surface area of HEA. In this regard, two main strategies were utilized. The first one is thermal etching with a chemical or gaseous agent and the second is blending with a second other materials. In this study, 23Fe−21Cr−18Ni−20Ti−18Mn HEA was synthesized via ball milling, and to our knowledge it was for the first time treated with HCl acid and used as an electrode material in SC structure. The selection of these five elements is based on their exceptional electrochemical properties. As d‐block elements, they are well‐known for their excellent electrochemical behaviour. This specific alloy composition has been employed to explore its electrocatalytic properties^[13]^ and its performance in supercapacitor applications. The obtained HEA and HEA treated with HCl (HEA‐T) were subjected to extensive characterization to understand their crystal structure, microstructure, surface area, and electrochemical properties. Although the crystal quality and the stoichiometric proportion of HEA almost remain the same with acid treatment, it triggered the specific surface area to enlarge due to the creation of numerous mesopores. The enlargement of the surface area led to more interaction between electrolyte and electrode, resulting in faster electron transfer, higher specific capacity, and cycling stability. Thus, these studies propose a favourable potential of HEAs as electrode material in SCs. The findings could pave the way for more efficient and sustainable energy storage solutions, addressing the growing global energy demands and supporting the transition to renewable energy sources.

## Materials and Methods

### Fabrication of Nano‐Structured 23Fe−21Cr−18Ni−20Ti−18Mn (HEA) Powders By Ball Milling

We have used Retsch Planetary Ball Mill PM 100 to fabricate HEA powders. Initially, the elemental powders such as Fe (99.6 % pure), Cr (99.8 % pure), Ti (99.6 % pure), Ni (99.5 % pure), and Mn (99.2 % pure) of composition 23Fe−21Cr−18Ni−20Ti−18Mn by wt.% were ball milled for 15 hours under toluene atmosphere. The ball‐to‐powder ratios were maintained at 6 : 1 with a mill speed of 300 rpm and the volume of the jar is 500 mL containing the 10 mm diameter chrome steel balls. Toulene was added to arrest the oxidation of the elements and also to aid the milling. Make sure to add toluene in such a way that must just immerse the balls and the elemental powder inside the jar. Ball milling was continued for 15 hours with 30 30‐minute breaks after every 30 minutes of milling; this was done to cool down the temperature of the jar. Generally, during ball milling, the continuous collision of balls‐jar‐powders results in increased temperature inside the jar and may result in phase transformation of the alloy. Therefore, we must allow the ball mill to cool down after every 30 minutes of milling. After 15 hours of ball milling, we have taken out the HEA powders.

### Treatment of 23Fe−21Cr−18Ni−20Ti−18Mn with HCl (HEA‐T)

HEA material was stirred in 1 M HCl acid solution at 500 rpm in a magnetic stirrer for 1 hour. The resulting solution mixture was separated by centrifugation at 7000 rpm and washed with distilled water until pH 7. The obtained paste particles were dried in a vacuum oven at 60 °C overnight.

### Materials Characterization

The HEA and HEA‐T crystal structures were characterized using an X‐ray diffractometer (XRD, Rigaku D/MAX‐2200) with Cu Kα radiation. The HEA and HEA‐T microstructures were characterized using a scanning electron microscope (SEM, TESCAN MAIA3 XMU). Chemical analysis was carried out using energy‐dispersive X‐ray spectrometry (EDS), EDS mapping. N_2_ adsorption‐desorption curves were recorded using an Autosorb‐iQ 2ST/MP instrument and the specific surface area was analyzed by the Brunauer‐Emmett‐Teller (BET) process.

### Electrochemical Characterization

To evaluate electrochemical features, HEA and HEA‐T electrodes were fabricated. Firstly, HEA and HEA‐T powders, super P (Carbon) and PVDF (polyvinidene fluoride) were mixed in a ratio of 8 : 1 : 1 in NMP (N‐Methyl 2‐Pyrrolidone) solvent, respectively, and mud‐like mixtures were obtained. After these mixtures were subjected to an ultrasonication process for 15 min., coated via the dip coating method on pre‐cleaned Ni foams (1x1 cm dimensions), and dried in a vacuum oven at 60 °C for 12 h. Electrochemical tests of HEA and HEA‐T electrodes were carried out using a ZIVE SP1 electrochemical workstation. Cyclic voltammetry (CV), Electrochemical impedance spectroscopy (EIS), and galvanostatic charge‐discharge (GCD) tests were carried out in a three‐electrode cell with Pt sheet as counter electrode, Ag/AgCl as reference electrode and fabricated electrodes as working electrode. 3 M KOH aqueous solution was used as an electrolyte medium in measurements.

## 2 Results and Discussion

**Figure 1:**
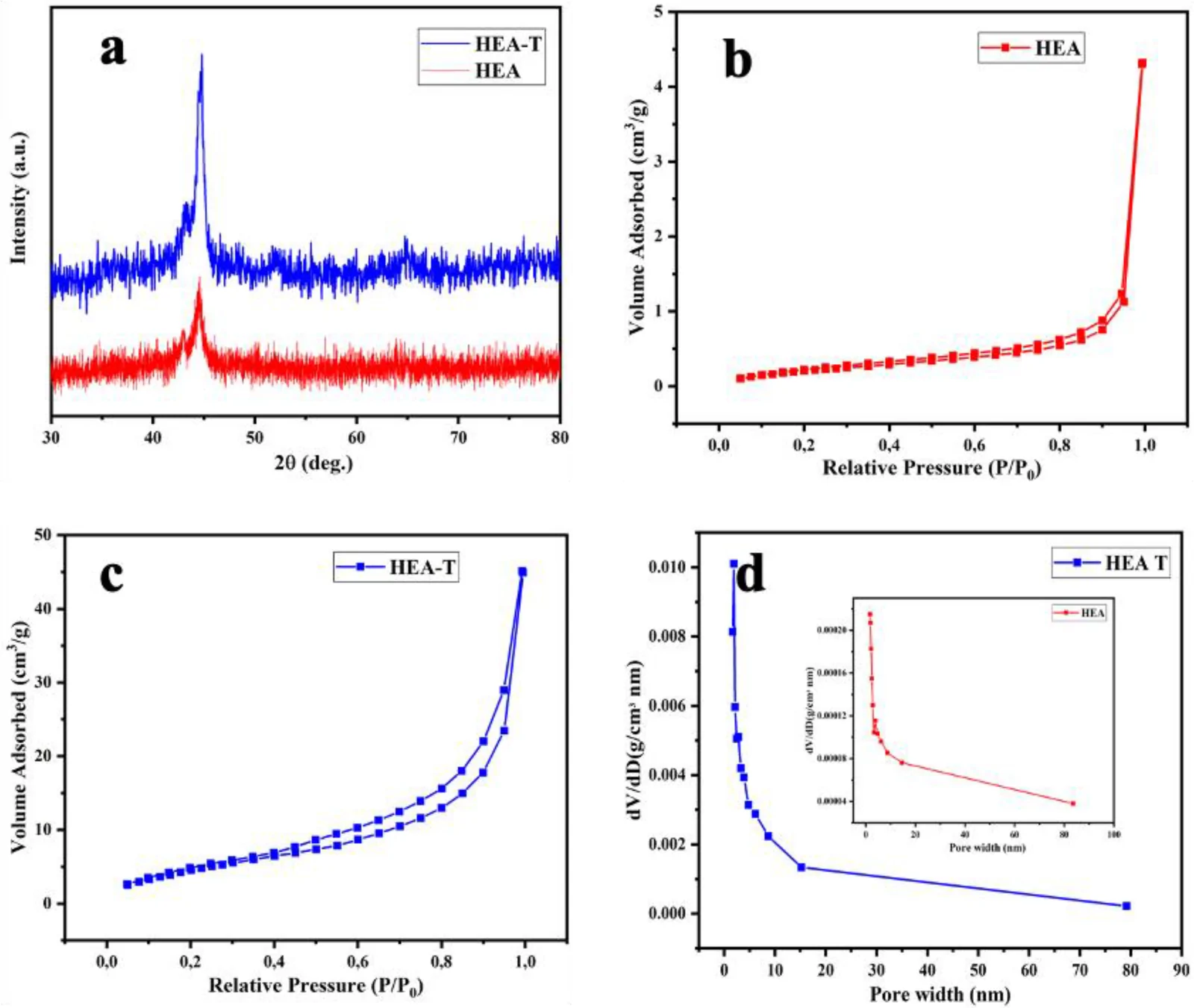
XRD patterns of HEA and HEA‐T (a), Nitrogen adsorption‐desorption isotherms of HEA (b) and HEA‐T (c), and pore size distribution curves of HEA‐T and HEA.

Figure 1a shows the XRD profile of HEA and HEA‐T samples. The diffraction peaks exhibiting (110), (111), and (200) X‐ray peaks at 43.08°, 44.75° and 64.79° respectively were identified as body‐centered cubic (BCC) phase.^[23]^ The XRD of HEA and HEA‐T confirms that Ni and Ti go into the interstitial regions of Fe. More importantly, treatment of HEA with HCl acid did not deteriorate the crystal lattice structure, and the corresponding diffraction peaks became more distinct and sharper, indicating that acid treatment improves the crystallinity.^[24]^ In addition, after acid treatment the content of BCC (2θ=44.75°) phase enhanced, leading to improvement of HEA stability at high temperatures.^[25]^ The specific surface area and pore features of HEA and HEA‐T were also investigated by N_2_ adsorption/desorption isotherms. As shown in Figure 1b–c, the adsorption/desorption curves belong to type‐IV patterns and show an H3 hysteresis loop, indicating that the prepared samples have mesoporous structure.^[26]^ Brunauer‐Emmett‐Teller (BET) specific surface area of the HEA and HEA‐T were determined as 0.859 and 18.72 m^2^ g^−1^, respectively. HCl treatment expanded the surface area about 20 times. Furthermore, the determined isotherms for HEA‐T had a more noticeable hysteresis loop at high P/P_0_ (0.4–1.0) than that of HEA, which exhibited a larger amount of mesopores on HEA‐T.^[27]^ To further investigate the effect of acid treatment on the surface morphology of HEA, pore size distribution curves of HEA and HEA‐T were obtained as in Figure 1d. It is observed that the pore size distribution around 5.0 nm of HEA‐T exhibited a huge increment compared to HEA due to converting the micropores to mesopores with acid treatment. Furthermore, the pore volume and radius are found to be for HEA 0.007 cm^3^ g^−1^ and 1.7 nm and for HEA‐T 0.069 cm^3^ g^−1^ and 1.9 nm. While the pore radius remains almost the same, the pore volume increased ~10 times with acid treatment of HEA, which indicated a considerable modification in the surface morphology of the HEA. The acid treatment created numerous mesopores on the surface of HEA, resulting in a larger specific surface area. Enlargement of the surface area possesses many advantages for electrode materials such as more interaction between electrolyte and electrode, fast electron transfer, and well diffusion electrolyte, resulting in enhancement of SC performance.^[28]^

**Figure 2:**
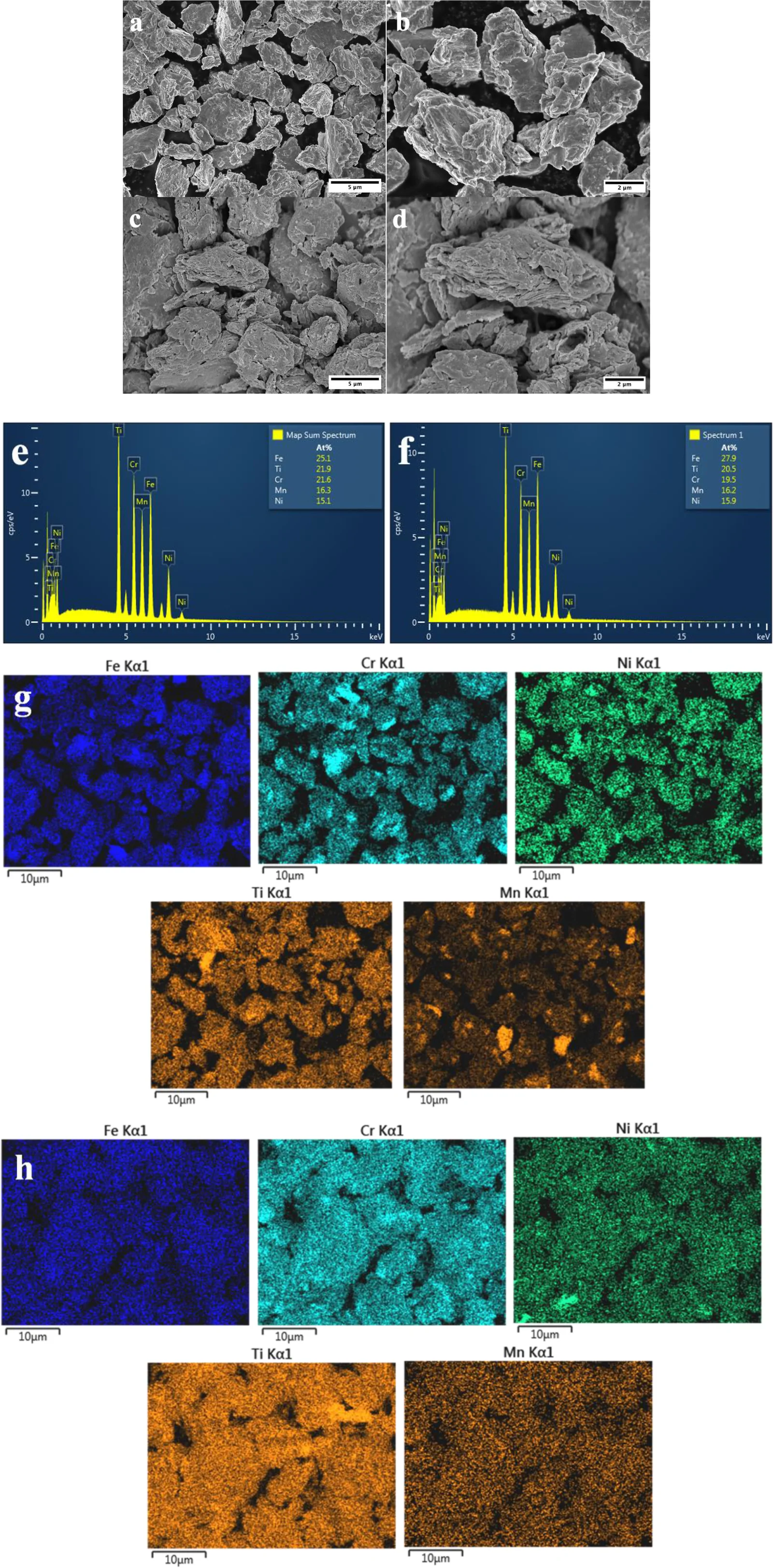
SEM images of HEA (a–b), and HEA‐T (c–d), EDX spectra of HEA (e) and HEA‐T (f), and EDS mapping of HEA (g) and HEA‐T (h).

Representative SEM images of HEA and HEA‐T powders were exhibited in Figure 2a–d. Untreated HEA powder demonstrated an agglomerated cluster‐like microstructure with a dimension of about 5 μm. Moreover, a relatively smooth surface was observed on the surface of the HEA and almost no pores could be seen. After HCl acid treatment (Figure 2b), plenty of pores were observed due to the generation of porous structure stemming from the reaction of HCl with HEA particles. Furthermore, the agglomerated HEA particles tended to expand and exfoliate to form sheet morphology. The creation of a lot of pores, tending to exfoliation and expansion resulted in a larger surface area of HEA‐T, which was consistent with the results of the BET analysis. These results reveal that the surface of HEA could be modified successfully by the strategy of acid treatment. To investigate the elemental composition and the effect of acid treatment on the composition of HEA, Energy Dispersive X‐ray Spectroscopy (EDX) tests were carried out as seen in Figure 2e–f. In the EDX spectra, peaks corresponding to Fe, Cr, Ni, Ti, and Mn elements were prominently observed and there is no contamination and oxidation appear in the HEAs. The intensity of each element was determined in proportion to the peak height, with elements exhibiting higher peaks being present in greater quantities within the alloy. The obtained HEAs exhibit a slightly high amount of Fe (at.%) in comparison to the ideal composition, due to the diffusion of Ti, Ni, Cr, and Mn into the crystal structure of Fe. A slight increment/decrement occurred in the concentration of certain elements after acid treatment. Furthermore, the stoichiometric proportion of HEA remained almost constant during the acid treatment due to a simultaneous etching reaction for different elements occurring at the same rate.^[29]^ The results demonstrated that treatment of HEA with HCl acid is an effective strategy to enlarge the specific surface area without changing the chemical composition of HEA. Furthermore, to evaluate the distribution of metal elements in HEA and HEA‐T, EDS mapping tests were conducted as shown in Figure 2g–h. It is obvious from the EDS mapping, that the distribution of constructive element of HEA‐T is more homogeneous than that of HEA, indicating better microstructure homogeneity of HEA‐T. These results are consistent with XRD and SEM analyses.

**Figure 3:**
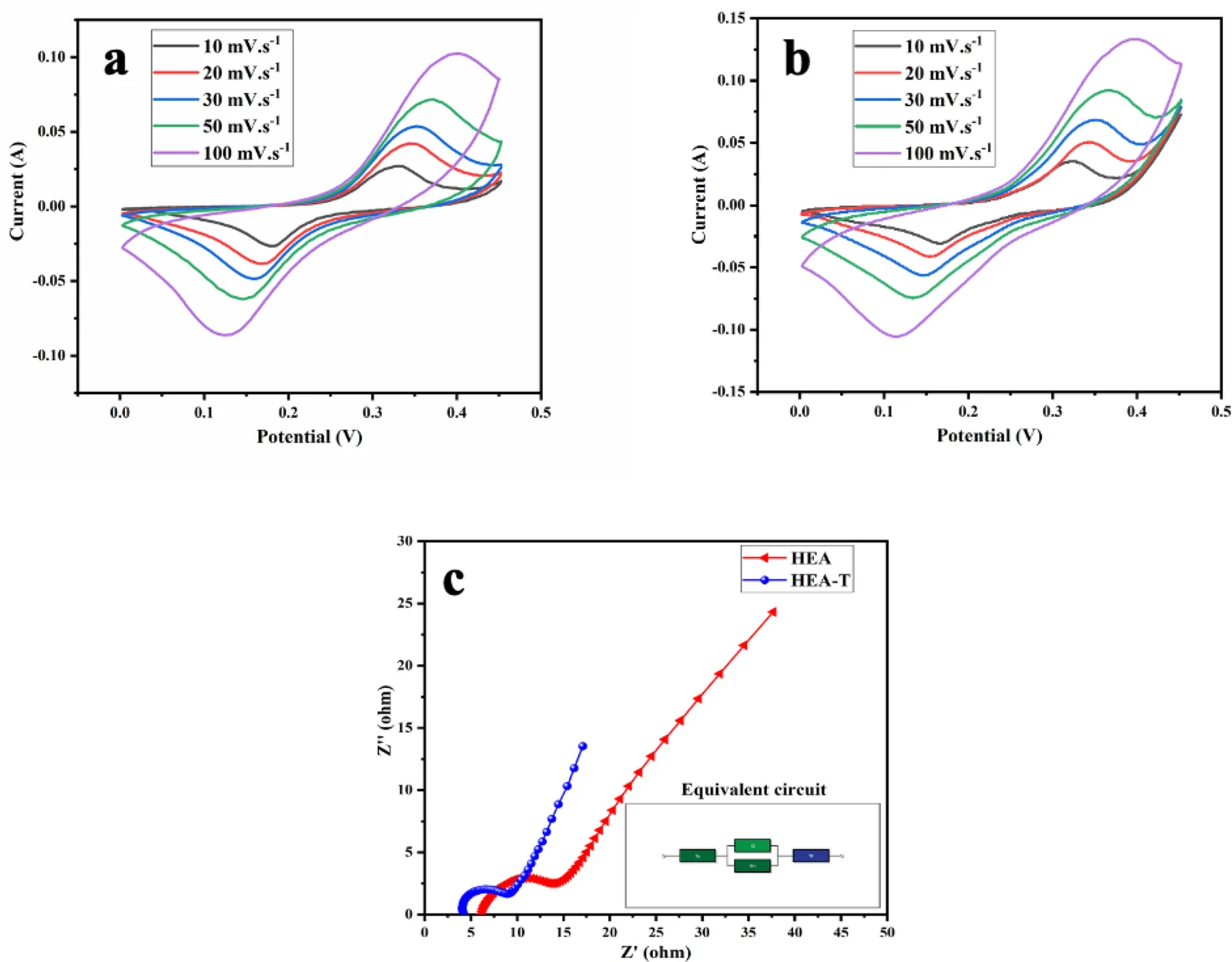
CV curves of HEA (a) and HEA‐T (b) at different scan rates, and Nyquist plots with equivalent circuit of fitting the Nyquist curves of HEA and HEA‐T electrodes (c).

To evaluate the electrochemical performances of HEA and HEA‐T electrodes, cyclic voltammetry (CV) tests were first carried out using 3.0 M KOH as the electrolyte in a three‐electrode system at various scan rates. The CV curves obtained at 10, 20, 30, 50, and 100 mV/s scan rates were exhibited in Figure 3a–b. The CV curves of both HEA and HEA‐T electrodes exhibited symmetrically redox peaks, demonstrating that the energy storage mechanisms of HEA and HEA‐T are pseudo‐capacitive characteristics and excellent reversible.^[16a]^ At the same scan rate, compared to the HEA electrode, the HEA‐T offers much greater current density and integrated area, indicating that electrocatalytic features of HEA‐T can be greatly enhanced by enlargement of the surface area.^[30]^ Furthermore, with the increase of scan rate, the current density boosts considerably, while the redox peak potential shifts to a more positive and negative potential side, demonstrating that such high electrocatalytic performance of electrodes.^[31]^ Moreover, with the increase in scan rate, the position of the redox peak in the CV curve of HEA‐T shifted more and the current density response increased more compared to HEA, probably due to the larger oxidative surface area of HEA‐T, resulting in a relatively lower resistance, higher charge transfer kinetics, and faster redox reactions at the interface of electrode/electrolyte. To further investigate the electrocatalytic properties of the fabricated electrodes, EIS tests were carried out in a frequency range of 0.01 Hz‐100 kHz at a potential amplitude of 10 mV. The obtained Nyquist plots of the HEA and HEA‐T electrodes were exhibited in Figure 3c. Nyquist plots exhibited a single semicircle in the high‐frequency regime and a curved line in the low frequencies. The obtained Nyquist curves were fitted with ZMAN software using an equivalent circuit. As a result, series resistance (R_s_), charge transfer resistance (R_ct_), and Warburg impedance (W) were found to be 6.08 Ω, 8.02 Ω and 179 mΩ for HEA electrode and 4.21 Ω, 4.87 Ω and 97 mΩ for HEA‐T electrode, respectively. The decrement in R_ct_ and R_s_ represents the increment of conductivity with acid treatment. Furthermore, a considerable decrease in W value exhibits the faster ion diffusion rate on the HEA‐T electrode compared to HEA. The improvement in ion diffusion rate can be due to the large surface area of the HEA‐T electrode, which led to more catalytic active surface area for interaction with the liquid electrolyte and generates numerous routes for ion diffusion between the electrode and the electrolyte.^[32]^

**Figure 4:**
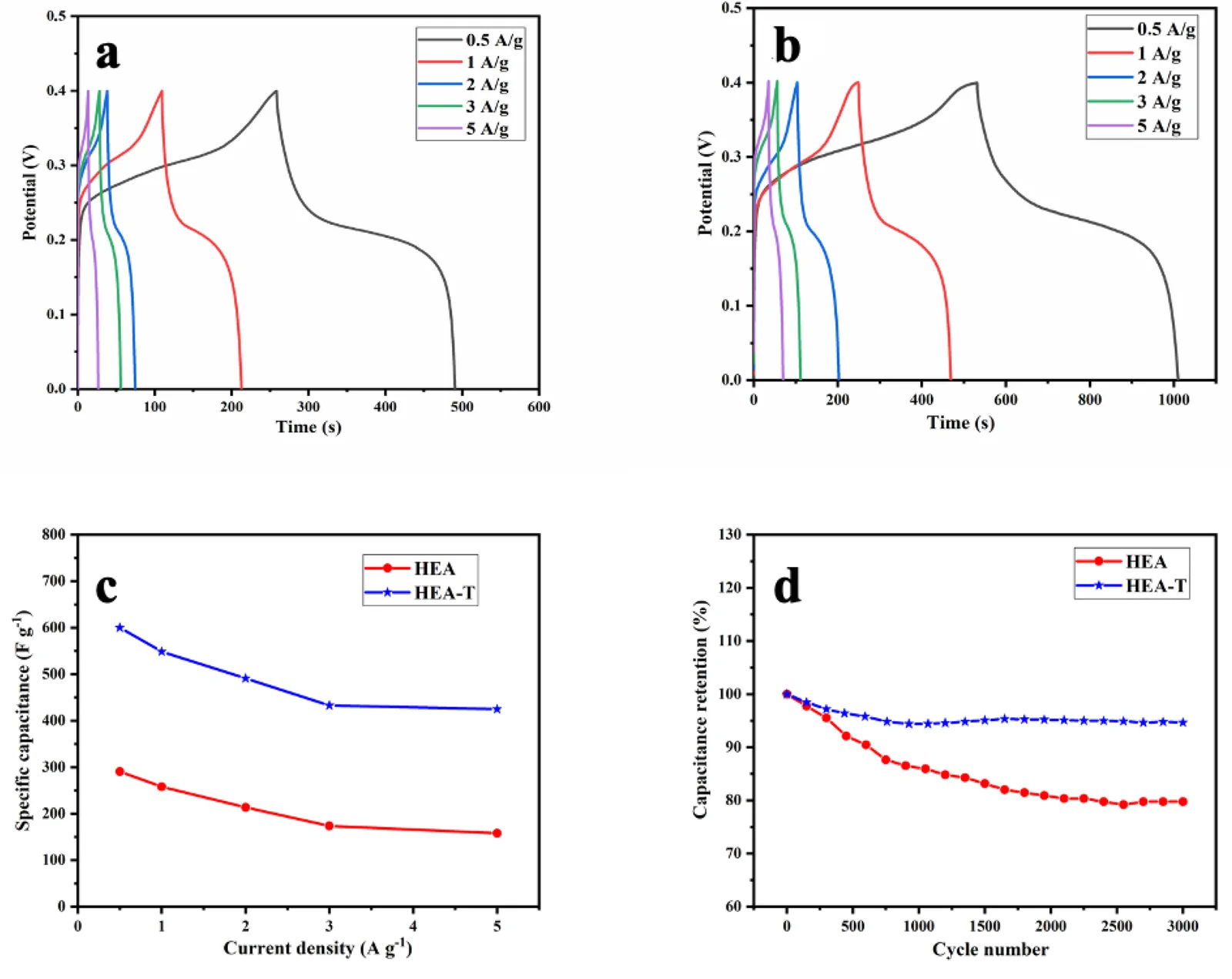
GCD curves of HEA (a) and HEA‐T (b) electrodes at different current densities, Specific capacitances of HEA and HEA‐T electrodes at different current densities (c), and Cycling performance of HEA and HEA‐T electrodes at a current density of 2 A g^−1^ (d).

Figure 4a–b presents the Galvanostatic Charge/Discharge (GCD) curves performed at 0.5 A/g, 1 A/g, 2 A/g, 3 A/g, 5 A/g current densities for HEA and HEA‐T electrodes. The specific capacitance (C_sp_) values were determined by the equation of $C_{sp}=\left(Ix\Delta t\right)/\left(mx\Delta V\right)$.^[33]^ Here, *“I*, *Δt*, *m*, and *ΔV* denote the discharge current, the discharge time, the weight of the HEA and HEA‐T loaded on the NF substrates, and the potential range, respectively. From GCD curves, C_sp_ values of the HEA and HEA‐T electrodes were estimated as 290.6 and 600 F/g, respectively at 0.5 A/g current density. With HCl acid treatment, the C_sp_ value of the HEA electrode was increased about 2.06 times due to the improvement of the electron transfer kinetics stemming from the enlargement of the active surface area.^[34]^ The enlargement of the electro‐catalytically active surface area possibly triggered the fast diffusion of electrolyte ions through the electrode material into the inner matrix, resulting in more interaction between electrolyte and electrode material.^[35]^ In addition, these enhancements in C_sp_ values can be attributed to the increase in the electron transfer rate and speed between electrolyte and HEA‐T. Furthermore, the variation of C_sp_ values with the change of current densities is exhibited in Figure 4c. When the current densities are increased, the shape and symmetry of the GCD curves remain identical, representing the stability of the electrodes. As shown in Figure 4c, the obtained C_sp_ values decrease with increasing the current density due to happening of charge‐discharge processes rapidly at higher currents. So, ions of the liquid electrolyte cannot diffuse deeply into the electrode material, resulting in insufficient redox reactions.^[36]^ With an increase of the current density from 0.5 A/g to 5 A/g, the specific capacitance HEA electrode decreased by approximately 46 %, while that of the HEA‐T electrode declined by only 29 %. This can be ascribed to the electrolytic ions that can penetrate in HEA‐T electrode more than of HEA even at high current densities, due to the sheet‐like morphology and larger pore volume of HEA‐T. For practical applications of SCs, capacity holding against charge‐discharge cycles is a crucial parameter to consider. Figure 4d shows the GCD cycling performance of HEA and HEA‐T electrodes which were carried out at 2 A/g current density. The capacitance retention of the HEA electrode decreases rapidly during the first few hundred cycles, and capacitance retention declines to 80 % and is not stabilized when the number of cycles increases to 3000 cycles, indicating the limited cycle life of the HEA electrode. In contrast to HEA, capacitance retention of HEA‐T electrode decreased slowly, and showed more stable performance (%95 stability), and stabilized over the number of cycles, demonstrating more durable, reliable, and long‐lasting electrode material for SCs.

## Conclusions

In brief, a high entropy alloy with the composition 23Fe−21Cr−18Ni−20Ti−18Mn was synthesized using a ball milling method and then subjected to HCl acid treatment to evaluate their potential in SC applications. Conducted tests reveal that HEA was successfully synthesized via the ball milling technique. Furthermore, the acid treatment led to an improvement in the crystal structure and microstructure of HEA. More importantly, by acid treatment, the specific surface area increased from 0.859 to 18.72 m^2^ g^−1^, and the micropore structure converted to mesopore sites leading to an enhancement of the pore volume from 0.007 to 0.069 cm^3^ g^−1^. As a result of these improvements, HEA‐T exhibited a tremendous energy storage performance and a more stable potential profile at high current densities compared to HEA. Furthermore, HEA and HEA‐T electrodes achieved C_sp_ of 290.6 and 600 F/g at a current density of 0.5 A/g, which is the C_sp_ of HEA‐T 2.06 times higher than that of HEA. The high specific capacitance values, stable potential profile, and ability to maintain performance even at high current densities make HEA‐T a promising candidate for SC applications.

## Conflict of Interests

The authors declare no conflict of interest.

## Data Availability

The data that support the findings of this study are available from the corresponding author upon reasonable request.
